# Multi-buffer zones reveal the relationship between spatial pattern of land surface temperature and land use indices in Guangzhou, China

**DOI:** 10.1038/s41598-026-44159-3

**Published:** 2026-03-19

**Authors:** Zhoujiang Liu, Kai He, Zehua Ke, Litao Yuan, Yanqin Mao, Yuning Zhang

**Affiliations:** 1 Institute of International Rivers and Eco-Security, Yunnan University, Kunming, China; 2 College of Surveying and Information Engineering, West Yunnan University of Applied Sciences, Dali, China; 3 Faculty of land and Resources Engineering, Kunming University of Science and Technology, Kunming, China; 4 College of Geography and Remote Sensing, HoHai university, Nanjing, China

**Keywords:** Land surface temperature, Land use indices, Land use, Pearson correlation coefficient, Concentric circles, Climate sciences, Ecology, Ecology, Environmental sciences

## Abstract

Land surface temperature is a crucial physical parameter in the examination of the natural ecological environment. The study utilized Landsat data to investigate land use indices and thermal environment change in Guangzhou, employing the radiative transfer equation, concentric circles, Pearson correlation coefficient, and other geospatial methods. Overall, as the distance from the city center increased, NDVI values tended to rise, while land surface temperature showed a gradual decreasing trend. Additionally, land surface temperature exhibited a negative correlation with NDVI and a positive correlation with NDBI. Barren had the highest LST, followed by impervious, while the water and the forest were cooler. The high-temperature area took on a V-shape, primarily situated in the west and southern areas, whereas the cooler temperature zone was mainly found in the northeast. The results can offer a scientific foundation for further exploration of the urban heat island formation mechanism, development of rational planning policies, and assessment of urbanization’s impact on local climate.

## Introduction

Land is the space carrier of human’s most basic social activities and ecological pattern construction^1^. It is crucial for sustaining the processes, functions, and structures of the ecosystem^2^. As the population grows and the range of human activities expands, the contradiction between man and land is becoming more and more prominent^3^. Land use change program is put forward, land use change research is becoming a hot spot^4^. Changes in land use illustrate the connection between humans and the land. The development and implementation of “human-land” coupling land use change simulation and application is an effective way to reveal the driving factors and evolution laws of land use^5^.

Land surface temperature (LST) is an effective indicator of earth-air interaction and an important factor affecting regional ecological environment^6^. The study of land surface temperature and its change can indirectly detect the status of urban surface heat island^7^. With the swift progress of urbanization and industrialization, the extensive expansion of artificial land surfaces has resulted in significant air pollution, which has triggered a range of ecological and environmental issues that pose direct risks to people’s lives and health. Quantitative inversion of spatial-temporal characteristics of LST is vital for future urban development planning and ecological protection. Remote sensing image data is widely used in the dynamic detection of LST because of its wide coverage and consistent temporal resolution. Various sensors, including AVHRR and MODIS, are currently employed in land surface temperature studies^8–11^. Due to the lack of resolution, it is more suitable for large-scale research^12^. The medium-resolution Landsat series data has a wider coverage and more abundant ground object information, making it more appropriate for estimating land surface temperature in mesoscale areas^13^.The Landsat data was widely used because of the easy availability of data. To illustrate this point, researchers compared three land surface temperature inversion methods using Landsat8 data^14^.

Land use change affects absorption rate of solar radiation, albedo, surface temperature, evaporation rate and heat exchange of soil, and also changes the greenhouse gas content in the atmosphere^15,16^. Land cover change can influence the change of vegetation to a great extent^17^. Human activities such as land degradation, urban expansion and deforestation will result in higher land albedo, reduced roughness, and the decrease of regional water cycling capacity, thus weakening the processes of transpiration, evaporation and precipitation processes, and contributing to an increase of LST^18^. Therefore, studying spatial-temporal pattern of LST, analyzing evolution law of urban thermal environment and the interaction relationship with land use can more objectively reveal the formation and mitigation mechanism of thermal environment, and provide reference for formulating urban ecological environment optimization policies^19^.

At present, in the study of the influencing factors of LST, the research is mostly focused on single factors^20–22^, with less emphasis on the development of a multi-factor simulation and analysis system. Zhang et al. found that the regulation effect of vegetation on land surface temperature was significantly different among different land use types, and presented nonlinear and threshold characteristics^23^. In the Bengaluru urban district, LST was negatively correlated with NDVI, while LST was positively correlated with NDBI. No significant correlation was found between LST and DEM despite elevation variations^24^. The correlation analysis method was used to investigate the statistical relationship between LST and NDVI, NDBI and NDWI^25^. Roohani Qadikolaei et al. revealed the important influence of geographical proximity on thermal environment differentiation^26^. Land cover change reflects the speed of urbanization, and surface temperature is an important indicator of urban heat island. However, studies that consider distance to explore surface temperatures are still scarce. Therefore, under the background of rapid urbanization of Guangzhou, the important city of Guangdong-Hong Kong-Macao Greater Bay Area, the study proposes a three-dimensional coupling of multi-buffer zone, multi-index, distance gradient, aiming to systematically reveal the variation characteristics of land surface temperature and key land use index with distance gradient, and provide a new analytical perspective for understanding the spatial heterogeneity of urban thermal environment.

## Materials and methods

### Study area

Guangzhou is situated in the south-central part of Guangdong Province, with an east longitude of 112°57’E-114°30’E and a north latitude of 22°26’ N-23°56’N. Integrating land transportation, air transportation and shipping, it is referred to as China’s “South Gate”^27^. With a long development history spanning over 2,000 years, it is a core city in the Guangdong-Hong Kong-Macao Greater Bay Area and the Pearl River Delta Economic Zone. It also serves as a central city along the “Belt and Road”^28^. Guangzhou has 11 districts: Liwan (LW), Yuexiu (YX), Haizhu (HZ), Tianhe (TH), Baiyun (BY), Huangpu (HP), Huadu (HD), Panyu (PY), Nansha (NS), Conghua (CH) and Zengcheng (ZC). The landscape is elevated in the northeast and lower in the southwest. Typical yearly temperature is about 20–22℃, the average annual rainfall ranges from 1229.6 to 2491.3 mm, and the average annual rainfall is generally exceeding 1500 mm. The area has undergone swift urban growth, marked by extensive urban land expansion and a considerable increase in the population moving into the city center over the past ten years.

**Fig. 1:**
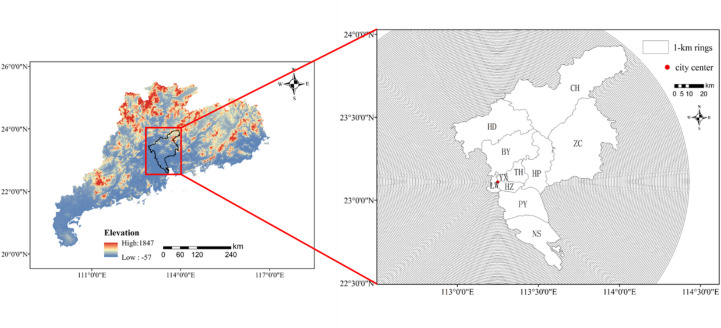
Study area.

### Data sources

The resolution of land use data used in this study is 30 m, and the time is the study period. The overall accuracy of this data set was over 80% (https://zenodo.org/records/12779975). Compared to other products, it has a higher temporal resolution^29^. This study utilized Landsat 8 data (https://espa.cr.usgs.gov/). The analysis involved the inversion of LST, NDVI, NDBI, and MNDWI, requiring two Landsat images to fully cover entire study area adequately. Cloudy and rainy weather is frequent in spring and summer in the study area, Landsat images are seriously polluted by clouds, and effective pixel coverage is insufficient; however, the atmospheric transparency is high from November to December, so continuous and high-quality Landsat products can be obtained, which ensures the spatial and temporal integrity of long-time series LST mapping. Therefore, all images were captured in mid-November or December to better reveal the relationship between land use and LST (Table 1).

**Table 1:** Data source.

Satellite	Sensor	Orbiter	Time	Resolution
Landsat 8	OLI/TIRS	122/42,122/43	2013-12-31	30 m (Band-8 excepted)
			2016-12-07	
			2019-11-14	
			2022-12-24	

### Methods

**Fig. 2:**
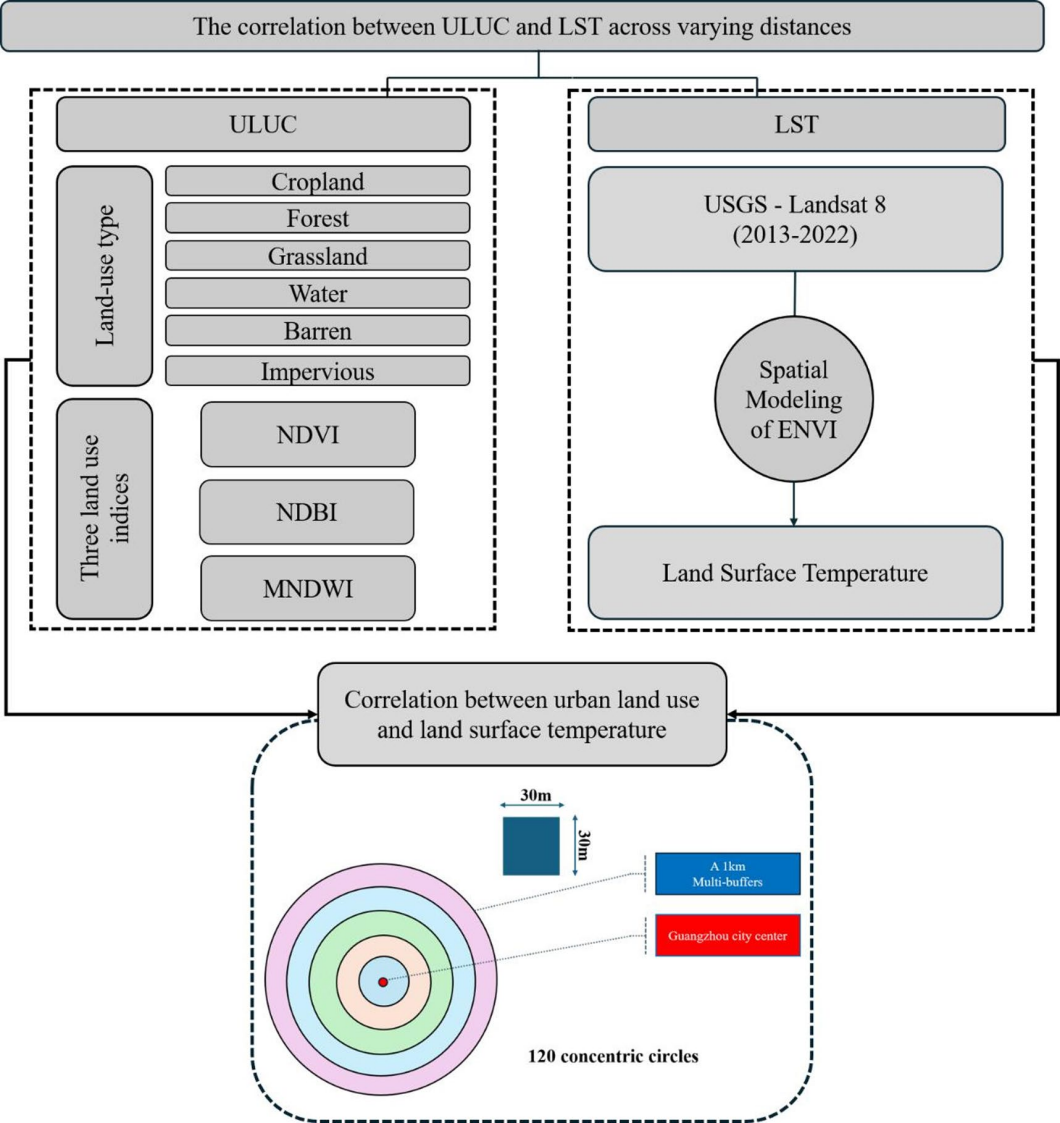
Flow chart of this study.

Land use data were combined with Landsat images to conduct land use classification, LST inversion, NDVI inversion, NDBI inversion, MNDWI inversion, and correlation analysis between LST and NDVI, NDBI and MNDWI. The city center of Guangzhou was buffered outward, forming 120 concentric circles with equal distance of 1 km. The radius of the city center of Guangzhou is also 1 km, as illustrated in Fig. 1. The following illustrates the research process (Fig. 2). We used the ArcGIS 10.8 for the study area and land surface temperature inversion mapping.

#### LST inversion

This study utilizes the radiative transfer equation method. While the process of LST inversion using this method is more intricate, its accuracy is higher. Where, the thermal infrared radiation brightness value L_λ_ collected by the satellite sensor can be expressed as (1).

1 $$L_{\lambda } = \left[ {\epsilon B\left( {T_{s} } \right) + \left( {1 - \epsilon } \right)L \downarrow } \right]\tau + L \uparrow$$

ε :Surface specific emissivity, Ts: True surface temperature K, B(Ts): Blackbody radiation. τ: Atmospheric transmittance.

When temperature is denoted as T, B(Ts) is calculated by the formula (2):

2 $$B\left( {T_{S} } \right) = \frac{{\left[ {L_{\lambda } - L \uparrow - \tau \left( {1 - \varepsilon } \right)L \downarrow } \right]}}{{\tau \varepsilon }}$$

Where Ts is calculated by the formula (3):

3 $${\mathrm{T}}_{\mathrm{s}}=\frac{{\mathrm{K}}_{2}}{\mathrm{ln}\left(\frac{{\mathrm{K}}_{1}}{\mathrm{B}\left({\mathrm{T}}_{\mathrm{S}}\right)}+1\right)}$$

In this experiment, the data source selected is Landsat 8 image of Guangzhou, and the corresponding sensor is OLI/TIRS. K_1_ = 774.89 W/m²·µm·sr and K_2_ = 1321.08 K.

#### Land use indices inversion

Normalized vegetation index (NDVI) serves as a key measure of vegetation growth state, and one of main indicators to measure the status and nature of regional ecological environment^30^. The quantitative relationship between vegetation index and LST has become an important field of urban thermal environment research^31^. The effectiveness of NDVI in evaluating and tracking global vegetation changes using satellite technology has been clearly proven^31,32^. NDVI is frequently utilized as a measure to determine the connection between vegetation and surface temperature^33^. It is expressed as (4):

4 $$NDVI=\frac{\mathrm{N}\mathrm{I}\mathrm{R}-\mathrm{R}\mathrm{E}\mathrm{D}}{\mathrm{N}\mathrm{I}\mathrm{R}+\mathrm{R}\mathrm{E}\mathrm{D}}$$

NIR: Near infrared band. RED: Infrared band.

Normalized Building Index (NDBI) is an index that represents the information of urban building land. It was put forward by domestic scholars on the basis of imitating NDVI. By utilizing this index, urban and other building land can be effectively extracted. The formula for estimating NDBI value using Landsat OLI/TIRS images is as follows (5):

5 $$NDBI=\frac{\mathrm{M}\mathrm{I}\mathrm{R}-\mathrm{N}\mathrm{I}\mathrm{R}}{\mathrm{M}\mathrm{I}\mathrm{R}+\mathrm{N}\mathrm{I}\mathrm{R}}$$

MIR: Mid-infrared band. NIR: Near-infrared band.

Modified Normalized Difference Water Index (MNDWI) is an index that signifies urban water information and can extract water details within the city. The formula for estimating MNDWI value using Landsat OLI/TIRS remote sensing images is as follows (6):

6 $$MNDWI=\frac{\mathrm{G}\mathrm{R}\mathrm{E}\mathrm{E}\mathrm{N}-\mathrm{M}\mathrm{I}\mathrm{R}}{\mathrm{G}\mathrm{R}\mathrm{E}\mathrm{E}\mathrm{N}+\mathrm{M}\mathrm{I}\mathrm{R}}$$

GREEN: Green band. MIR: Mid-infrared band.

#### Correlation analysis

Pearson correlation coefficient (PCC) was used to characterize the relationship between LST and NDVI, NDBI and MNDWI. It involves examining variables related to each influencing factor and calculating PCC between each influencing factor and the research object to assess the strength of their relationship^34^. PCC can be calculated by the formula (7).

7 $${\mathrm{R}}_{\mathrm{x}\mathrm{y}}=\frac{{\sum}_{\mathrm{i}=1}^{\mathrm{n}}\left({\mathrm{x}}_{\mathrm{i}}-\acute{y}\right)\left({\mathrm{y}}_{\mathrm{i}}-{\acute{y}}\right)}{\sqrt{{\sum}_{\mathrm{i}=1}^{\mathrm{n}}\left({\mathrm{x}}_{\mathrm{i}}-{\acute{x}}\right){\sum}_{\mathrm{i}=1}^{\mathrm{n}}\left({\mathrm{y}}_{\mathrm{i}}-\acute{y}\right)}}$$

R_xy_: PCC between the two variables.

x_i_ ${\mathrm{y}}_{\mathrm{i}}$: the pair of observations. $\acute{x}$: the mean NDVI, NDBI or MNDWI. $\acute{y}$: the mean LST. n: the number of paired observations.

## Results

### Distribution pattern of land surface temperature

To quantitatively examine the spatial distribution features of land surface temperature in Guangzhou, this research categorized LST. It has been shown that the mean standard deviation(STDEV) method is superior to the isometric method in expressing the spatial distribution and details of temperature^35^. Therefore, it was adopted in the study to divide LST from 2013 to 2022 into five levels. The detailed classification criteria can be found in Table 2.

**Table 2:** LST classification standards.

Detailed Zoning	Conditions*
Low LST (LTZ)	Tb < µ - std
Sub low LST (SLTZ)	µ - std ≤ Tb < µ − 0.5std
Medium LST (MTZ)	µ -0.5std ≤ Tb ≤ µ + 0.5std
Sub high LST (SHTZ)	µ + 0.5std < Tb ≤ µ + std
High LST (HTZ)	Tb > µ + std

*In the table, µ is mean LST, std is STDEV of LST.

**Fig. 3:**
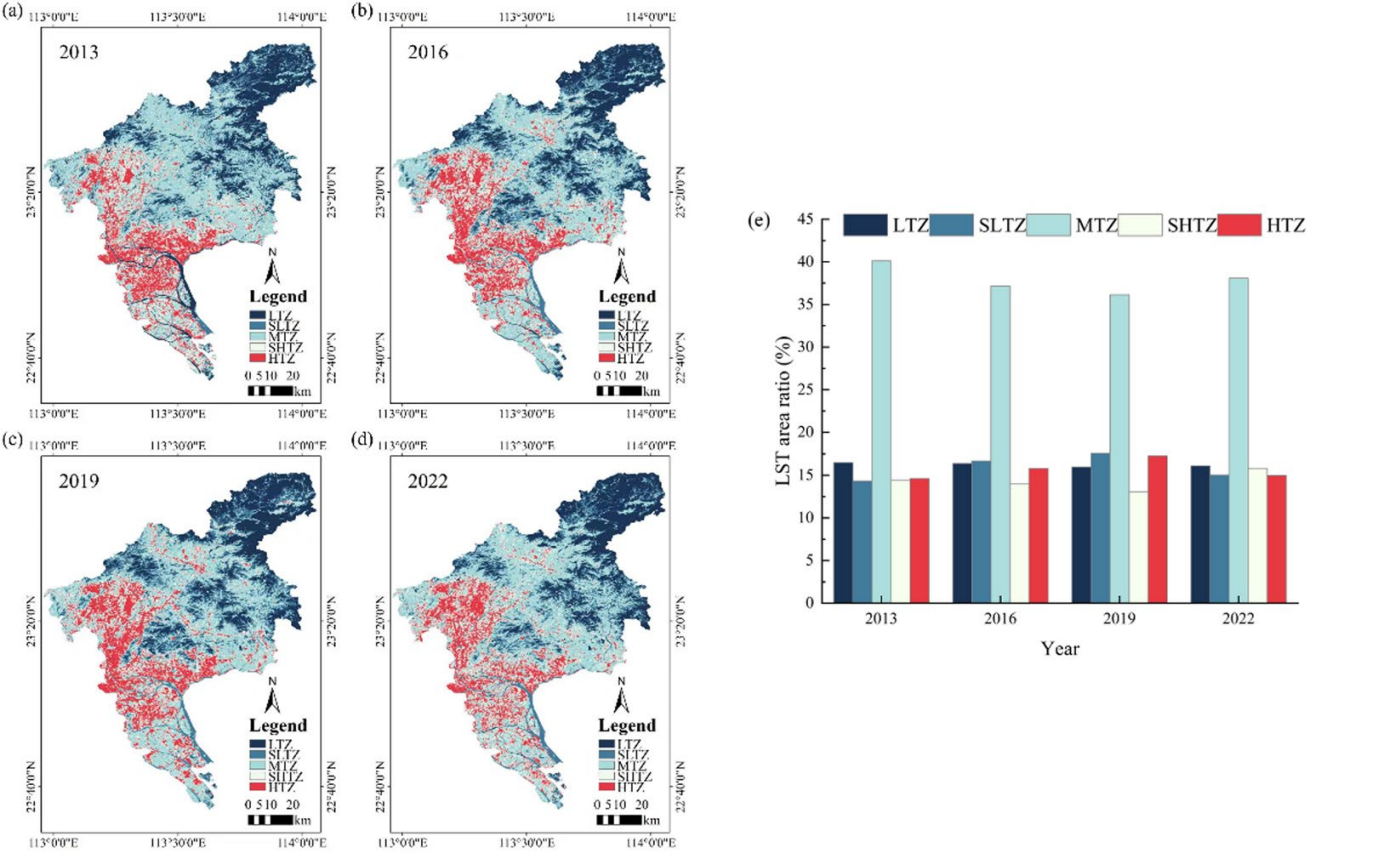
Spatial-temporal distribution and area proportion of each temperature class in study period.

It can be observed from the map that HTZ area takes on a V-shape, primarily situated in the mid-west and southern areas. LST in the city center was significantly higher than that in the surrounding areas (Fig. 3). HTZ was predominantly located in the central and western regions, while LTZ was mainly found in the central and eastern regions as well as northwest regions. Urban heat island (UHI) exhibited the characteristic of large region concentration in space. This distribution of LST in the region was primarily attributed to the prevalence of urban land and major traffic arteries in the urban central region. Characteristics of building materials and human activities could contribute to the relatively high land surface temperature in these areas. Conversely, low-temperature areas were primarily situated in the northeast, where vegetation was abundant, and human activities had minimal impact, the land surface temperature was relatively low. In the four periods, the medium temperature region always occupies the first place (Fig. 3). It was worth noting that the high temperature area was gradually increasing, rising from 14.6% in 2013 to 17.3% in 2019, and slowing down from 2019 to 2022.

Land surface temperature was calculated using Landsat images for 2013, 2016, 2019 and 2022. Then the average surface temperature of each concentric circle was calculated. As shown in Fig. 5a–d, in all four periods, the mean LST was decreasing from urban centers to suburbs. Within 10 km from the city center, there was a relatively large fluctuation, which increased first and then decreased, which may be caused by the uneven distribution of urban land. It decreases significantly from 10 km to 40 km and continues to decline to a sustainable level after 40 km. In addition, we found that after 40 km, Guangzhou built-up area showed a large area of decrease, which was an important reason for the surface temperature to stabilize after this threshold. On the whole, the difference of Rural-urban surface temperature showed a gradual rising trend during 2013–2022.

The mathematical models of land surface temperature and urban center distance in 2013, 2016, 2019 and 2022 were linear regression models. The corrected coefficient of determination of the four models was greater than 0.94. The concentric circle average LST fluctuated with distance from the city center. The results showed that the mean LST of concentric circles was significantly related to the distance from the city center (Fig. 5a–d). Additionally, it indicated that all four linear regression models exhibited negative slopes. This suggested that LST steadily declines from the urban core to the suburban regions between 2013 and 2022.

**Fig. 4:**
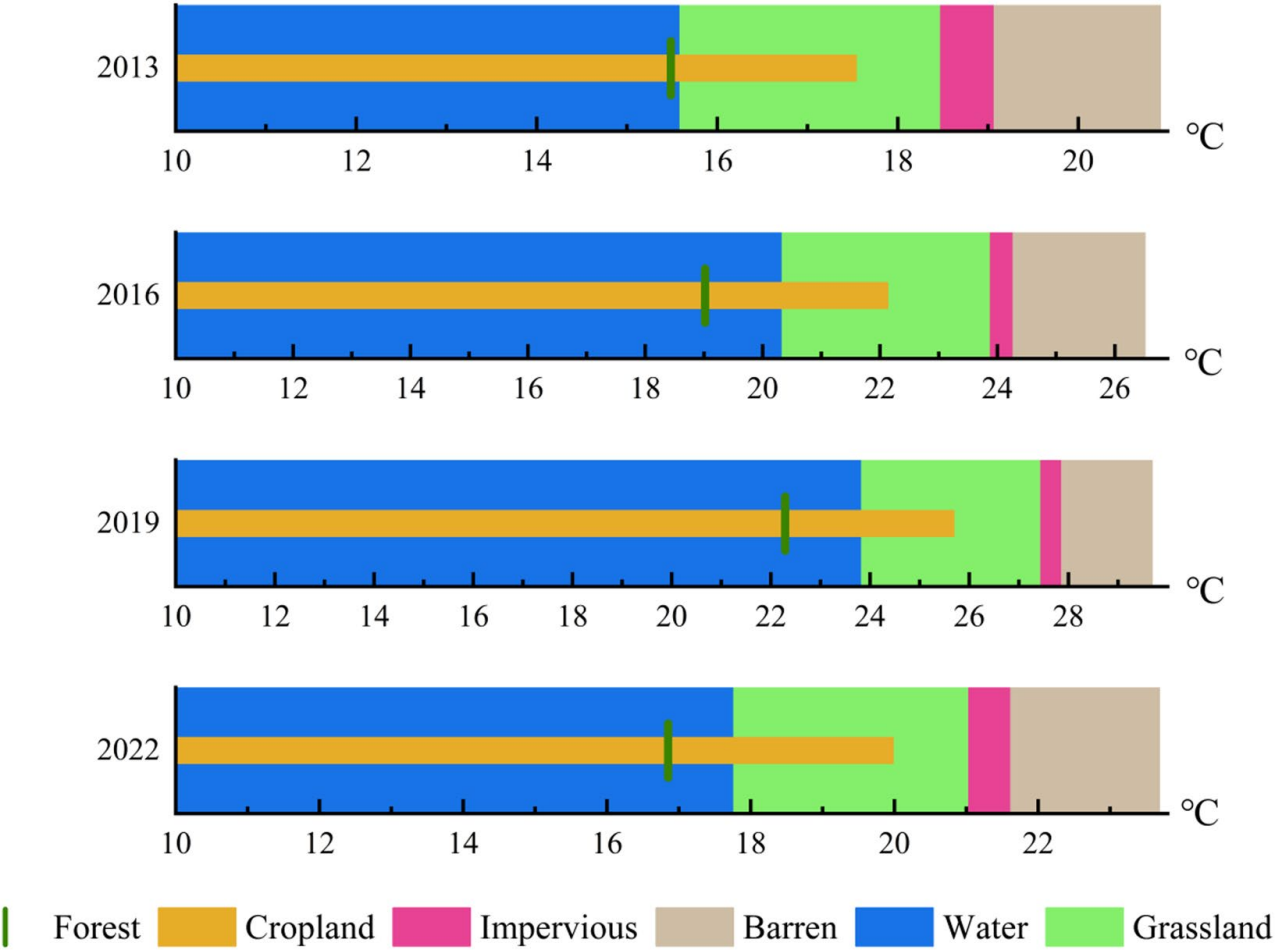
Average LST values of different land use from 2013 to 2022.

As depicted in Fig. 4, the average temperature rise rate of impervious surfaces ranks second after barren areas. The magnitude of change in grasslands and forests was similar, possibly due to differences in plant growth. Moreover, from 2013 to 2022, the mean LST of different land use types followed the order: Barren > Impervious > Grassland > Cropland > Water > Forest.

Four classes of calculated values of LST for each land use type were presented in Table 3 to assess their effects on LST. Cropland and Water exhibited the smallest total STDEV. In contrast, impervious areas showed a larger STDEV. Grassland and Forest also demonstrated high STDEV because of the presence of shrubs and trees, which may result in a broad temperature range. Barren land, characterized by its intricate nature, displayed a wide range of temperature variations, which may lead to a high STDEV as well.

**Table 3:** Land surface temperature differences among different land classes during 2013–2022.

Year	LST (℃)	Cropland	Forest	Grassland	Water	Barren	Impervious
2013	MIN	-8.118	-3.807	10.333	4.166	4.833	-8.987
	MAX	35.377	37.116	31.050	37.110	28.521	37.116
	MEAN	17.538	15.486	18.477	15.571	20.906	19.053
	STDEV	1.296	1.505	2.389	1.693	2.008	1.664
2016	MIN	-3.588	1.374	5.729	-0.948	-2.116	-2.554
	MAX	40.356	41.047	38.095	36.899	35.734	41.047
	MEAN	22.128	19.021	23.887	20.307	26.508	24.244
	STDEV	1.517	1.692	3.147	1.637	2.534	1.864
2019	MIN	15.314	14.059	18.158	17.051	15.346	11.409
	MAX	44.126	43.859	37.000	44.324	36.590	44.245
	MEAN	25.684	22.288	27.449	23.799	29.679	27.835
	STDEV	1.612	1.799	2.605	1.632	2.753	1.778
2022	MIN	-13.314	-3.137	0.070	-2.020	-6.138	-12.355
	MAX	39.611	40.955	30.286	39.970	31.630	40.955
	MEAN	19.979	16.850	21.038	17.745	23.682	21.598
	STDEV	1.668	1.966	3.137	1.835	4.601	1.980

### Land use indices

**Fig. 5:**
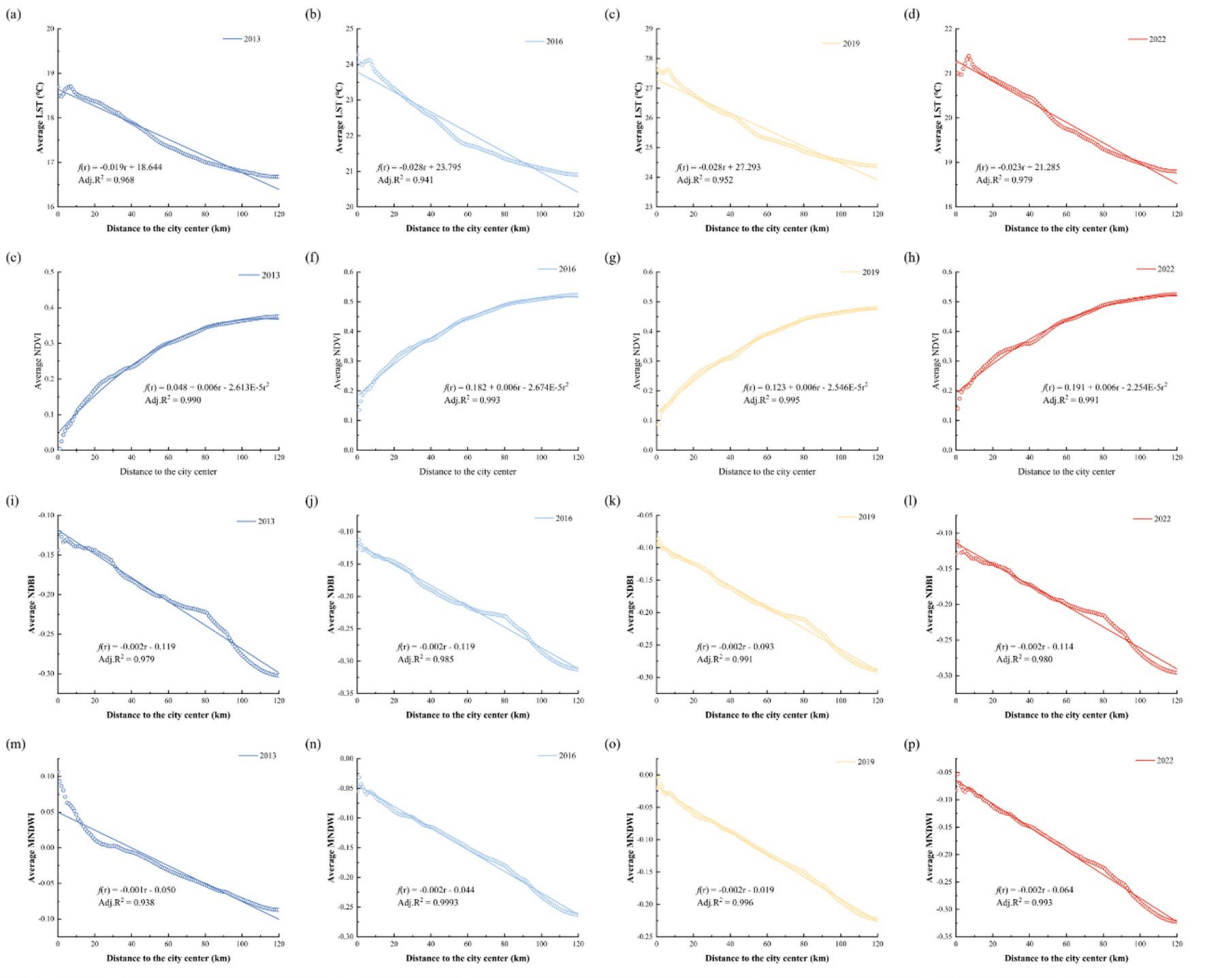
Variation of LST and land use indices with distance from city center.

The NDVI was calculated using Landsat images from 2013, 2016, 2019 and 2022. As illustrated in Fig. 5e–h, over the four periods, the mean NDVI showed a consistent increase from urban centers to suburban areas. The rate of increase was higher within the 0–40 km, accelerated further between 40 and 50 km from the city center, and continued to rise steadily to a sustainable level beyond 50 km. Figure 5e–h showed the relationship between NDVI and distance simulated by a second-order polynomial model. The adjusted coefficient of determination for all four models exceeds 0.99.

As depicted in Fig. 5i–l shows, in the four periods, the mean NDBI showed a trend from urban centers to suburban areas. It exhibited a gradual decrease within the 0–30 km range, followed by a more rapid decline between 30 and 80 km from the city center, and a further accelerated decrease beyond 80 km. Figure 5i–l illustrated that the distances between mean NDBI and urban center for the years 2013, 2016, 2019, and 2022 can be represented by linear regression models. The corrected coefficient of determination of the four models was greater than 0.97. The mean concentric circle NDBI fluctuated with distance from the city center. Additionally, they had negative slopes. This indicated that the mean NDBI gradually decreases from urban center to suburban area during 2013–2022.

The mean MNDWI decreased from urban centers to suburban areas. In 2013, the mean value of MNDWI decreased rapidly from 0 to 20 km from the center of Guangzhou, changed slowly from 20 to 40 km, and decreased at a faster rate after 40 km. From 2016 to 2022, there was a relatively large fluctuation within 10 km from the city center, and decline rate was basically the same after 10 km. Figure 5m–p showed that the mathematical models of mean MNDWI and the distance from urban centers in 2013, 2016, 2019 and 2022 are linear regression models. The corrected coefficients of determination of the four models are greater than 0.93.

### Correlation analysis between LST and land use indices

Connection between LST and land use indices (NDVI, NDBI, and MNDWI) was investigated. Following the principle of uniform distribution, a total of 100 random points were generated in various classes each year, with a minimum of 30 m between points to match the spatial resolution of remote sensing images (resulting in a total of 2400 points). Subsequently, pixel values of NDVI, NDBI, MNDWI and LST were extracted, and respective PCC were calculated.

**Fig. 6:**
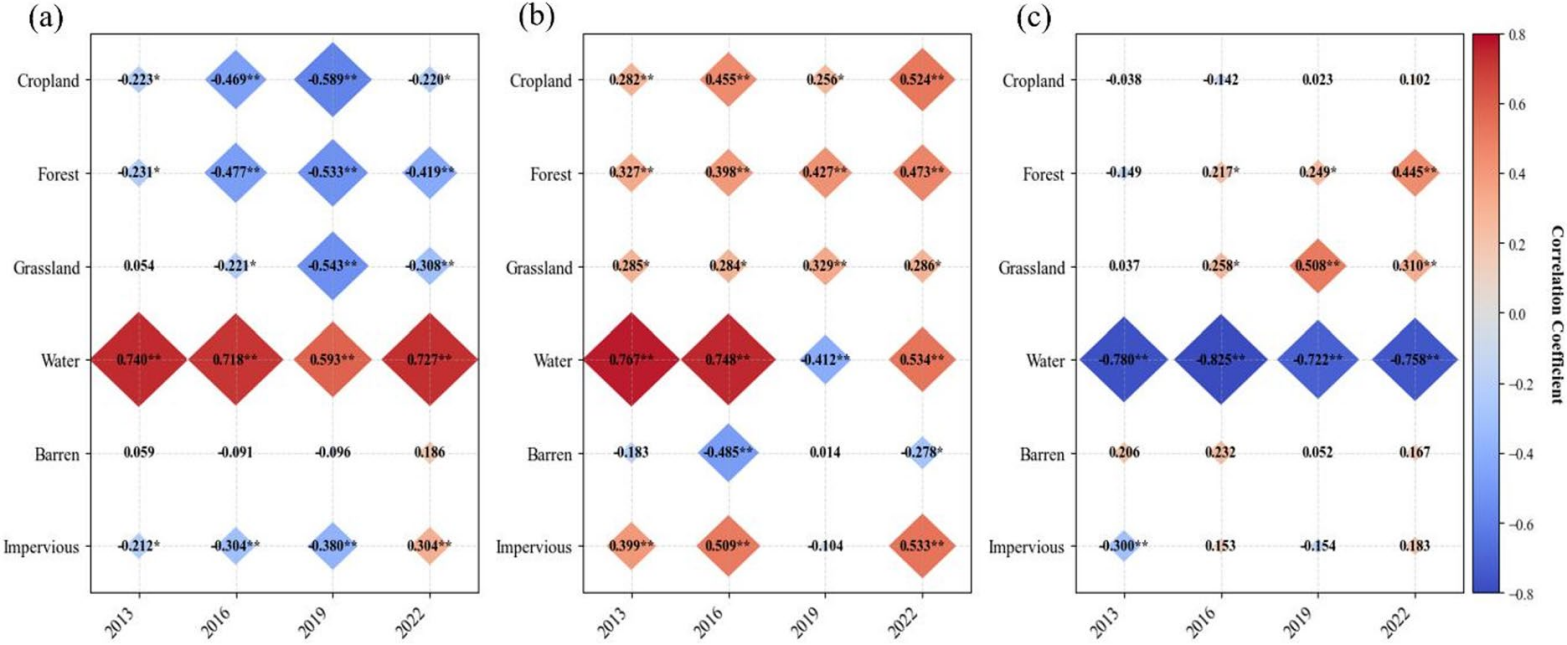
Pearson coefficients of LST and NDVI (**a**), NDBI (**b**), MNDWI (**c**) in different land-use types. *p < 0.05 **p < 0.01.

In various land use types, the NDVI of forest had a strong negative correlation coefficient with LST, and cropland was similar to forest. The findings showed a significant relationship between NDVI and LST, with an inverse relationship between the two variables (Fig. 6a). As the NDVI increased, the LST could decrease.

The positive PCC between NDBI and LST was stronger, reaching 0.533 in the impervious areas (Fig. 6(b)). The negative PCC between barren and LST was weak. The findings showed a significant connection between NDBI and LST. Specifically, as NDBI rose, LST also tended to rise, indicating a positive relationship between the two. Expanding the area of impervious could intensify and contribute to the expansion of UHI.

The highest PCC observed between MNDWI and LST was 0.825 (for Water in 2016) as indicated in Fig. 6(c), which demonstrated a negative correlation. Increasing the area of water body can reduce the surface temperature appropriately and alleviate the urban heat island effect.

## Discussion

We found that the proportion of high-temperature areas has been continuously increasing from 2013 to 2019. This may be due to the implementation of urban planning, green space or other environmental measures. The results of this study show that there was a negative correlation between LST and NDVI and a significant positive correlation with NDBI when considering distance from the city center, which was consistent with other local studies^36–38^. However, interestingly, the proportion of high-temperature areas decreased from 2019 to 2022, which to some extent indicated that the urban heat island in Guangzhou had not been increasing year by year. To alleviate the urban heat island in Guangzhou, attention should be paid to the planning and construction of land use. The relationship between LST and land use indicates that water and forests have lower temperatures compared to other land use types. This is mainly because water have a higher specific heat capacity and their temperature changes slowly. Guangzhou has a subtropical Marine monsoon climate with abundant sunlight and heat. After the water surface is heated, the heat can be transferred from the surface to the deep layer or dispersed over a larger area through water flow, waves and vertical convection, thus avoiding the accumulation of heat on the surface. In the urban center area and in the forest, the root systems of trees absorb soil moisture and consume a large amount of latent heat during the transpiration of leaf stomata, effectively reducing the temperature of the plants and the surrounding air^39^. Paddy fields, as the main type of cultivated land in Guangzhou, have water covering their surfaces. The cooling effect of paddy fields is close to that of water bodies. The soil structure of paddy fields that are meticulously cultivated will be more conducive to water retention^40^. Due to the characteristics of materials such as asphalt and concrete, the impervious surfaces have a low albedo and a high heat capacity, which can retain more heat and result in persistently high temperatures at night^41^. As an economically developed city in China, Guangzhou had dense floors that impede ventilation, making it difficult for heated air to diffuse. Heat was stored between the streets and high-rise buildings. In the subsequent urban planning, priority should be given to urban ventilation corridors^42^. The establishment of wetland parks^43,44^, green parks^45,46^, and the optimization of the drainage system in urban areas have a positive effect on alleviating the small-scale urban heat island in the city center. The study have shown that building green roofs can reduce the daytime air temperature by 0.4 degrees Celsius^47^.

There were two limitations in this study. First, the accuracy of the land use data used could not really reflect the actual accuracy of land use classification in Guangzhou City. Regional land-use classification based on high-precision remote sensing images, and improving the accuracy of land-use classification on the region are the following research needs to be considered. Second, since the retrieval of LST in this study was based on Landsat data, it may be due to cloud cover and atmospheric disturbance affecting the accuracy of LST, which will also affect the correlation analysis between LST and land use indicators at high temporal resolution. In future research, we should consider the fusion of multiple sensors, field measurements of surface temperature and machine learning to improve the accuracy of LST inversion.

## Conclusion

Taking Guangzhou City as the study area, the correlation between NDVI, NDBI, MNDWI and the spatiotemporal distribution of land surface temperature was analysis. There was an obvious urban heat island effect in Guangzhou. HTZ areas were mainly concentrated in the west of Guangzhou, as well as the southern. These were areas with lots of impervious surfaces, and the land surface temperature was relatively high. LTZ was primarily found in the northeast, characterized by vegetation, which may lead to relatively lower land surface temperature. The mean LST of barren areas was the highest, with impervious coming in second. Green roofs, ventilation corridors and constructed wetlands were recommended to be given priority in high and sub-high temperature zones, or in the core area of 40 km from the city center.

## Data Availability

The data that support the findings of this study are available from the corresponding author, upon reasonable request.
